# Thermotaxis of Human Sperm Cells in Extraordinarily Shallow Temperature Gradients Over a Wide Range

**DOI:** 10.1371/journal.pone.0041915

**Published:** 2012-07-25

**Authors:** Anat Bahat, S. Roy Caplan, Michael Eisenbach

**Affiliations:** Department of Biological Chemistry, The Weizmann Institute of Science, Rehovot, Israel; University of Hull, United Kingdom

## Abstract

On the basis of the finding that capacitated (ready to fertilize) rabbit and human spermatozoa swim towards warmer temperatures by directing their movement along a temperature gradient, sperm thermotaxis has been proposed to be one of the processes guiding these spermatozoa to the fertilization site. Although the molecular mechanism underlying sperm thermotaxis is gradually being revealed, basic questions related to this process are still open. Here, employing human spermatozoa, we addressed the questions of how wide the temperature range of thermotaxis is, whether this range includes an optimal temperature or whether spermatozoa generally prefer swimming towards warmer temperatures, whether or not they can sense and respond to descending temperature gradients, and what the minimal temperature gradient is to which they can thermotactically respond. We found that human spermatozoa can respond thermotactically within a wide temperature range (at least 29–41°C), that within this range they preferentially accumulate in warmer temperatures rather than at a single specific, preferred temperature, that they can respond to both ascending and descending temperature gradients, and that they can sense and thermotactically respond to temperature gradients as low as <0.014°C/mm. This temperature gradient is astonishingly low because it means that as a spermatozoon swims through its entire body length (46 µm) it can sense and respond to a temperature difference of <0.0006°C. The significance of this surprisingly high temperature sensitivity is discussed.

## Introduction

When human spermatozoa become capacitated they acquire a number of properties that, together, confer on them fertilizing ability [1]. One of these properties is the ability to be thermotactically active [2]. This property is manifested by the ability of the capacitated spermatozoa to change their swimming direction according to a temperature gradient (towards the warmer temperature) [3], [4]. The molecular mechanism underlying this process appears to involve the phospholipase C (PLC) signaling pathway, in which inositol 1,4,5-trisphosphate (IP_3_) production results in the opening of IP_3_ receptor Ca^2+^ channels and, consequently, in Ca^2+^ release from internal stores. This leads sequentially to modification of the flagellar bending and swimming patterns [5]. In spite of this information, a number of basic questions are still open, such as: How wide is the temperature range in which human spermatozoa are thermotactically responsive? Does this range include an optimal temperature to which spermatozoa are attracted, or do spermatozoa always swim within this range towards the warmer temperature? Accordingly, can spermatozoa sense and respond to descending temperature gradients or do they only respond to ascending gradients (as was proposed for the chemotactic response of sea urchin spermatozoa to the chemoattractant resact [6])? What is the minimal (threshold) temperature gradient to which they can thermotactically respond? Our aim in this study was to resolve these questions.

## Results

### The Effective Temperature Range of Thermotaxis

**Figure 1:**
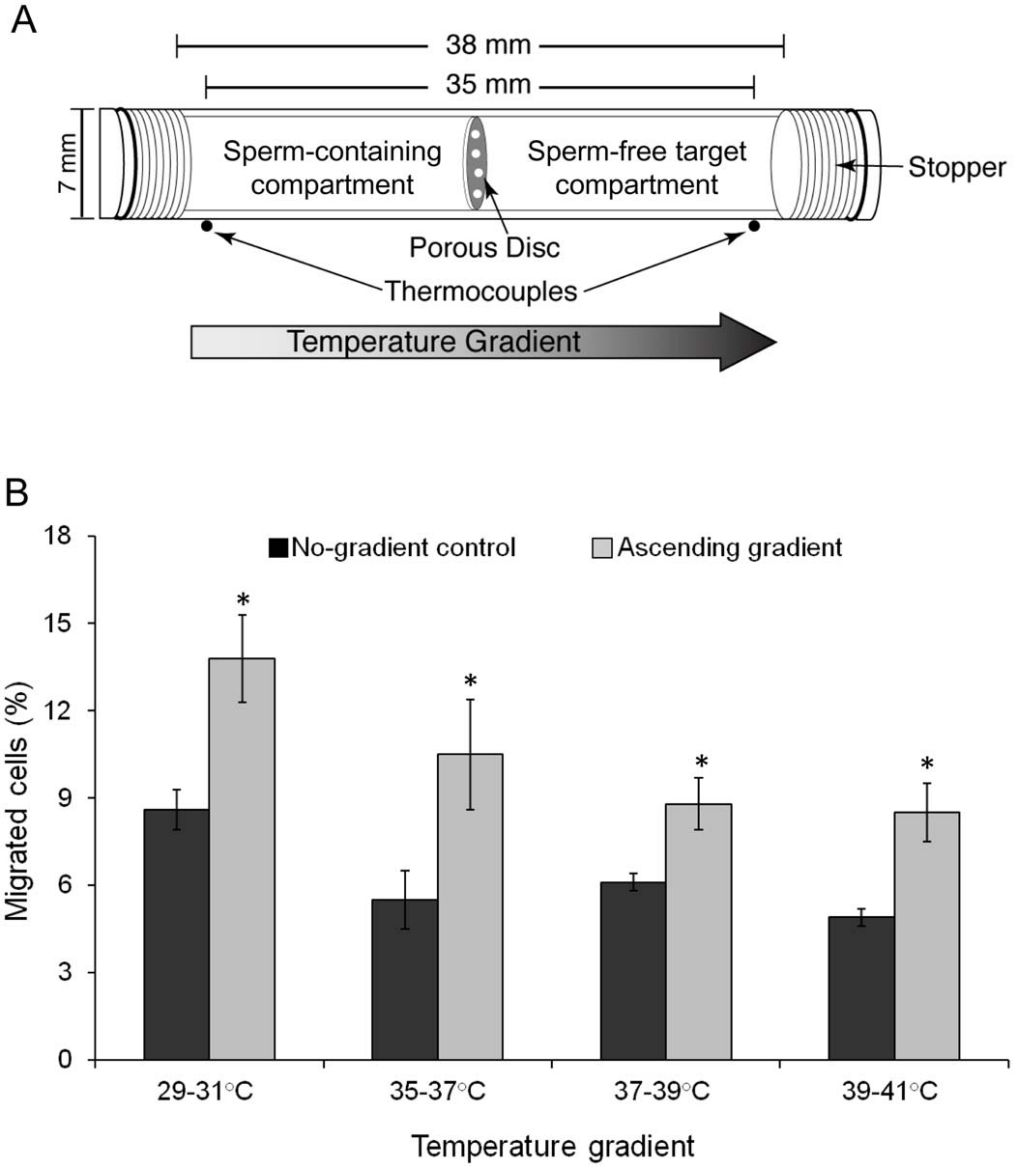
Migration of human spermatozoa in an ascending temperature gradient. **A:** Schematic illustration of the Lucite tube composed of two compartments for the separation process [5]. Thermocouples at both ends of the tube holder measured the temperatures at these locations. The two compartments were separated by a thin disc (316 stainless steel) having pores, 40 µm in diameter. **B:** Migration at various temperatures. The results are the mean ± SEM of 6–19 determinations (3–4 experiments for each temperature gradient tested). The temperatures shown in the abscissa were those measured by the thermocouples at both ends of the tube (externally to the tube). Asterisks above the columns indicate a statistically significant difference from the respective no-gradient control (*P*≤0.02, according to Student’s *t*-test).

To determine the temperature range in which thermotaxis is effective, we measured the migration of spermatozoa, pre-allowed to capacitate, from the cooler compartment of a thermoseparation tube (Figure 1A; see also [5]) to the warmer one. In all cases the temperature difference between the external thermocouples at both ends of the tube (Figure 1A) was 2°C, shifted over a relatively wide temperature range. Clearly, thermotaxis (i.e., the difference between the gradient and the control) was effective in the whole measured range (Figure 1B).

### The Preferred Temperature in this Range

**Figure 2:**
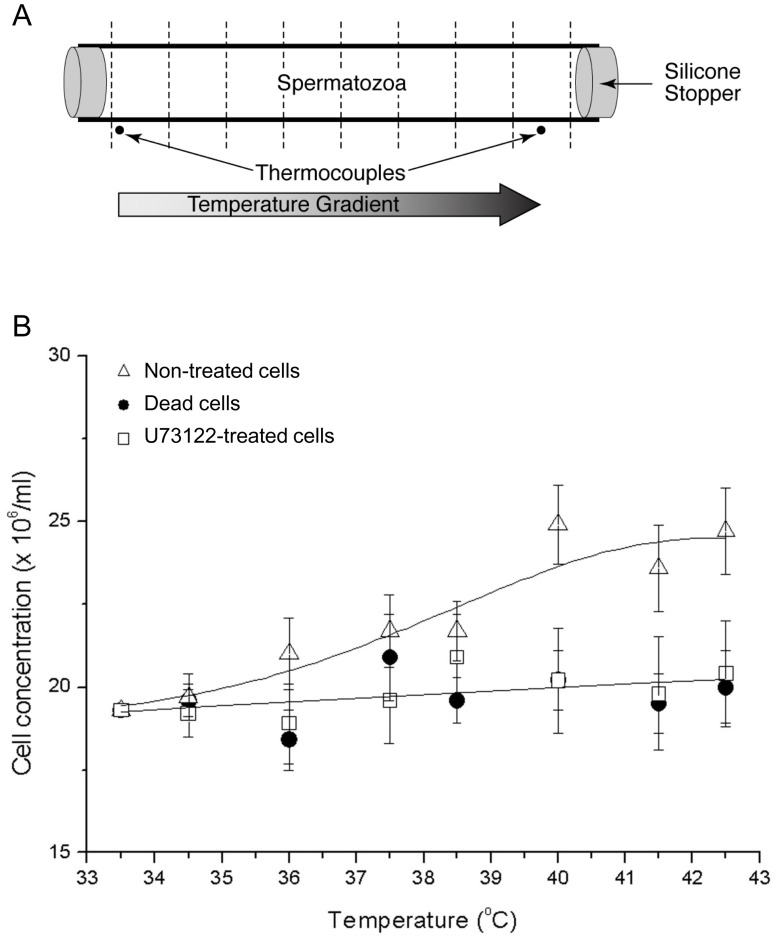
Human sperm distribution in a linear temperature gradient. **A:** Schematic illustration of the drinking straw, fitted to the dimensions of the thermoseparation device, and used to measure the sperm distribution in a linear temperature gradient. The straw, homogeneously filled with human spermatozoa at the commencement of a measurement, was frozen in liquid nitrogen at the end of the measurement and cut into 8 equal parts at the locations marked by dashed lines. **B:** Temperature preferences of human spermatozoa. The temperatures shown in the abscissa were measured inside the straw. The number of spermatozoa in the straw’s outermost segment (the warmer side) could not be counted due to loss of volume (since this was the segment cut last). For this reason the highest temperature shown in the figure is 41.7°C rather than 42.3°C. The results with the inhibitor U73122 were corrected for the solvent (ethanol) effect by subtracting the difference between ethanol and untreated cells (control) from the values obtained with U73122 at each temperature tested. Both the results (mean ± SEM) of untreated cells (9 experiments, 20 measurements in total) and the results of cells treated with U73122 (7 experiments, 13 measurements) were normalized according to the results of dead cells (4 experiments, 12 measurements) at 36.8°C. Sperm accumulation of untreated cells at 40.4°C, 41.2°C and 41.7°C was significantly higher than sperm accumulation at 36.8°C, 38.1°C, 38.5°C, 38.9°C and 39.7°C (*P*<0.0001, according to the contrast *t*-test). The connecting line is a hypothetical sigmoidal-curve fit (R^2^ = 0.91; Origin 6.1 software, OriginLab). The differences between the untreated cells and the negative controls (dead and U73122-treated cells) were statistically significant only at temperature values ≥40°C (Marked with Asterisks; *P*≤0.04, according to the one-way ANOVA). The straight line was calculated according to the average of the negative controls at each tested temperature.

To determine the accumulation preferences of human spermatozoa we examined whether they tend to accumulate at a certain temperature or whether they always swim up the temperature gradient. We put a drinking straw (Figure 2A), containing a uniform sperm concentration throughout, in a thermoseparation device that maintains a linear temperature gradient [5]. We exposed the spermatozoa (pre-allowed to capacitate) in the straw for 20 min to a linear gradient from 36.8±0.4°C to 42.3±0.4°C (±SEM, measured inside the straw and verified for linearity – see Materials and Methods and Figure S1). We then quickly froze the straw in liquid nitrogen, cut it into equal segments, and counted the number of spermatozoa in each segment (excluding the last one at the warmer side due to technical reasons). Evidently, the sperm distribution in the straw changed, with the sperm concentration shifting to the warmer temperatures (Figure 2B). More spermatozoa accumulated at T ≥40°C than at any other temperature tested (Figure 2B), suggesting that temperature values ≥40°C are thermotactically preferred by human spermatozoa. All the experimental points could be best fitted to a sigmoidal (Gaussian) curve (R^2^ = 0.91). This empirical choice seemed preferable to a linear fit (R^2^ = 0.89) since on *a priori* grounds the curve would be expected to reach saturation. The motility parameters of the cells at the various temperatures were not significantly different (Table 1).

**Table 1:** Kinetic parameters of human sperm motility under the conditions of the accumulation assay^a^.

Temperature (°C)	VCL (µm/s)	VSL (µm/s)	STR (%)	LIN (%)	MOT (%)	HYP (%)	Total number of analyzed cells
33.5	70±7	37±3	79±4	55±9	59±4	5±1	2939
34.5	77±9	40±3	78±5	54±8	61±6	8±4	2442
36	72±5	39±5	78±5	55±9	59±5	5±3	2716
38.5	80±2	45±5	79±3	57±8	53±4	6±4	3501
40	85±4	48±5	79±3	57±7	61±3	5±3	3428
41.5	85±2	51±7	79±4	59±9	52±5	4±3	3357
42.5	85±2	52±6	80±3	60±8	58±4	5±4	2831

a The sperm samples contained 3.5% PVP. The results are the mean ± SEM of 3 experiments (each being the average of duplicate determinations carried out for 80 s). The significance of the difference between temperatures was tested for each parameter by one-way ANOVA with Tukey-Kramer Multiple Comparisons post-test and found insignificant.

We repeated the temperature dependence measurements with cells that had been pre-treated with U73122 (a specific PLC inhibitor that has been shown to inhibit sperm thermotaxis [5]) as a control for non-thermotactic accumulation, and with dead non-motile cells as a control for passive accumulation due to physical forces (e.g., convection or temperature-dependent diffusion). Both controls appeared to be temperature-independent (Figure 2B). The observation that the difference between the experiment and the controls was only significant at the warmer part of the straw (≥40°C; Figure 2B) is consistent with the conclusion, made above, that human spermatozoa prefer warmer temperatures within the tested range.

### Response to a Descending Temperature Gradient

**Figure 3:**
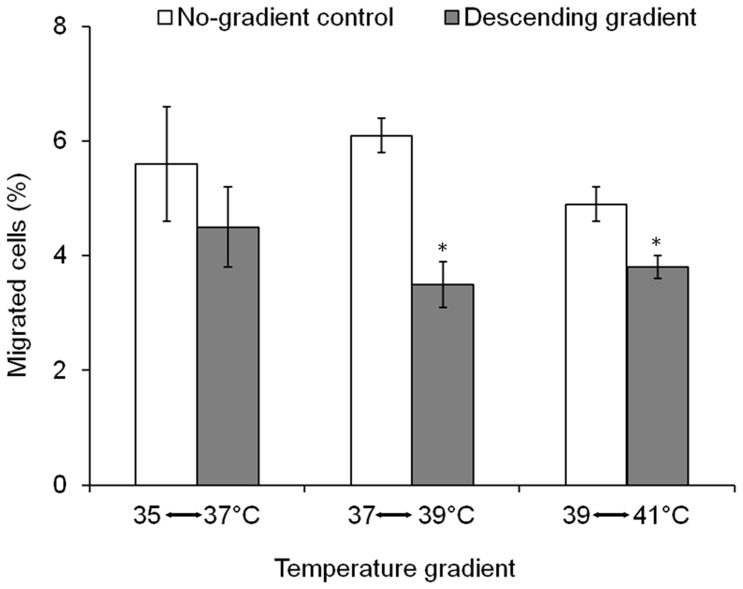
Migration of human spermatozoa in a descending temperature gradient. The temperatures shown in the abscissa were measured by the thermocouples at both ends of the tube (externally to the tube). The results are the mean ± SEM of 6–19 determinations (4 experiments for each temperature gradient tested). Asterisks above the columns indicate a statistically significant difference from the respective no-gradient control (*P*≤0.01, according to Student’s *t*-test).

The preference of human spermatozoa for warmer temperatures (Figure 2B) and their tendency to swim up the temperature gradient [3], raises the question of whether the spermatozoa at all sense and respond to descending temperature gradients. To address this question we compared the sperm accumulation between two configurations: a descending gradient in which the temperature of the sperm-free compartment (the target compartment) was 2°C lower than that of the sperm-containing compartment, and a no-gradient control (ΔT = 0°C, reflecting random swimming) in which the temperatures of both compartments were the same (the higher temperature of the two, for example, the temperature in the no-gradient control for the gradient 37–39°C was 39°C). The spermatozoa in both configurations were allowed to capacitate prior to the experiment. In the descending gradient, the accumulation was slightly or significantly lower (depending on the temperature range) than in the control (Figure 3). We also examined other temperature ranges, 29–31°C, 33–37°C and 30–40°C. In all of them we had similar observations (data not shown). The lower accumulation relative to the control suggests that capacitated spermatozoa do respond to a descending temperature gradient. Whether or not they actually sense the decrease in temperature remains to be seen (see Discussion).

### The Minimal Gradient to which Spermatozoa can Respond Thermotactically

**Figure 4:**
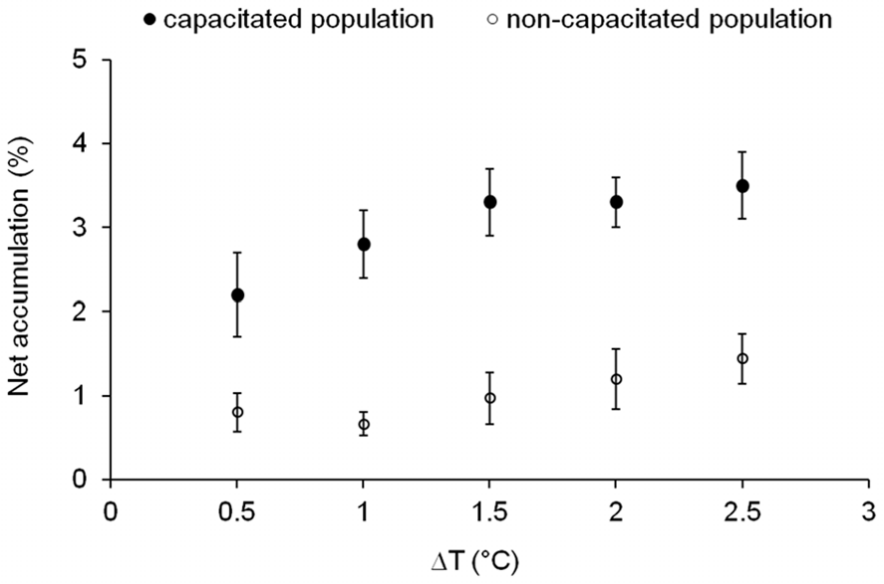
Dependence of sperm accumulation on the magnitude of the temperature difference. The temperature of the sperm-containing compartment was 36.5°C in all runs. The temperature differences shown in the abscissa were measured by the thermocouples at both ends of the tube (externally to the tube). Net accumulation was calculated by subtracting the no-gradient control accumulation from the sperm accumulation in a temperature gradient. The results are the mean ± SEM of 7–9 determinations (3–5 experiments). The difference between capacitated and non-capacitated sperm is very significant (*P*<0.0001, according to one-way ANOVA with Tukey-Kramer Multiple Comparisons post-test).

To determine the minimal thermal gradient (i.e., threshold gradient) to which human spermatozoa can respond, we measured their migration from the sperm-containing compartment of the thermoseparation tube (Figure 1A) to the sperm-free target compartment under a number of temperature gradients. We kept the temperature of the sperm-containing compartment at 36.5°C in all runs, and the temperature of the target compartment at a different constant temperature, varying between the runs from 37 to 39°C (with 0.5°C intervals). Thus, over the 3.5 cm distance between the thermocouples, the temperature difference varied from 0.5 to 2.5°C. As a negative control, both compartments were maintained at the same temperature (36.5°C). The temperature dependence of the response of the spermatozoa (pre-allowed to capacitate) seemed to be a saturation curve, with saturation achieved at 1.5°C difference (Figure 4). Here, too, the motility parameters of the cells at the various temperatures were not significantly different, except for VSL (Table 2). (We also studied the opposite configuration in which we kept the temperature of the sperm-free target compartment at 41°C in all runs, while the temperature of the sperm-containing compartment varied from run to run between 36 and 40°C. The results were qualitatively similar [Figure S2].) Technically, we could not reliably carry out measurements at temperature differences lower than 0.5°C. However, the observation that at ΔT = 0.5°C the sperm response was still substantial (Figure 4) suggests that human spermatozoa can respond even to temperature differences lower than 0.5°C. Taking into consideration the distance between the thermocouples, this further suggests that human spermatozoa can respond to temperature gradients lower than 0.014°C/mm.

**Table 2:** Kinetic parameters of capacitated human sperm motility under the conditions of the thermotaxis assays^a^.

Temperature (°C)	VCL (µm/s)	VSL (µm/s)	STR (%)	LIN (%)	MOT (%)	HYP (%)	Total number of analyzed cells
36	97±8	42±1	71±4	46±4	65±4	7±3	4036
37	95±4	48±2	74±4	51±5	68±4	4±2	3966
38	100±7	47±2	72±3	49±4	66±6	5±2	3494
39	101±6	51±1*	73±3	51±3	65±1	4±1	3980

a The sperm samples did not contain PVP. The results are the mean ± SEM of 3 experiments (each being the average of duplicate determinations carried out for 80 s).

* A significant difference (*P*<0.01) relative to 36°C, as determined by one-way ANOVA with Tukey-Kramer Multiple Comparisons post-test.

To verify that the observed sperm accumulation was due to thermotaxis, we measured the accumulation of sperm samples that had not been allowed to capacitate. Since only capacitated spermatozoa are apparently thermotactically responsive [3], the anticipation is that non-capacitated spermatozoa would not accumulate. Indeed, the accumulation of these control sperm samples (containing only 0.9±0.6% capacitated spermatozoa [±SEM; n = 5 samples]) was significantly lower (Figure 4; *P = *0.0002) than the accumulation of the experimental samples (containing 7.6±2.0% capacitated spermatozoa [n = 5]), even though the motilities of the experimental and control samples were similarly high (data not shown).

## Discussion

In this study we made a number of observations, some of which are quite unexpected. Perhaps the most challenging observations are the ability of human spermatozoa to respond thermotactically to extremely shallow temperature gradients and to do it over a relatively wide temperature range, wider than the range considered physiological. Other surprising observations are that human spermatozoa appear to seek warmer temperatures even when the warmer temperature is beyond the temperature range believed physiological, and that they respond not only to ascending temperature gradients but also to descending ones. These findings have important bearings on the physiology of sperm behavior, discussed below.

### The Measured Sperm Accumulation is the Result of Thermotaxis

As shown for human and rabbit spermatozoa, sperm thermotaxis is demonstrated by changing the direction of swimming according to the temperature gradient [3]. However, in this study we measured sperm accumulation in a thermoseparation device rather than changes in swimming direction, mainly because the long tube in the accumulation assays enabled us to measure sperm response to much shallower temperature gradients. Even though sperm accumulation can be the outcome of processes other than taxis, primarily temperature-dependent changes in swimming speed (thermokinesis), temperature-dependent passive diffusion and trapping of various kinds [7], accumulation in the thermoseparation tube was shown to faithfully reflect thermotaxis [5]. With respect to thermokinesis and passive diffusion, it should be pointed out that these processes, which reflect the effect on cell movement of absolute temperature rather than a gradient of temperature, result in behavior opposite to that observed here. Instead of accumulation in the warm zone, depletion occurs. This is because spermatozoa move faster at higher temperatures and, therefore, the rate of leaving the warm zone is greater than the rate of entering it. In bacteria this may even provide a safety mechanism [8]. The following lines of evidence provide further assurance that the measured accumulation was the outcome of thermotaxis. First, cells treated with U73122, shown earlier to inhibit thermotaxis without affecting motility [5], lost their ability to accumulate in the warmer temperature (Figure 2B). Likewise, non-motile cells did not accumulate (Figure 2B), excluding passive causes of accumulation (e.g., by temperature-dependent diffusion or convection). Second, non-capacitated spermatozoa, known to be thermotactically non-responsive [3], essentially did not accumulate in the warmer compartment (Figure 4). Third, sperm accumulation in an ascending temperature gradient was higher than in a no-gradient control (Figure 1B) and lower in a descending gradient (Figure 3), as the sperm accumulation criterion for taxis requires [7], [9]. And fourth, the accumulation was not due to trapped hyperactivated spermatozoa at the higher temperature, as the measured level of hyperactivated spermatozoa was similar at all measured temperatures (Tables 1 and 2). This appears to contradict an earlier study [10], which described preferred sperm accumulation at 40°C relative to 23°C due to a higher level of hyperactivated spermatozoa. The difference between the observations of both studies is, probably, due to the different experimental conditions: a more viscous medium in our experiments due to the presence of PVP, the use of fresh (in our study) rather than thawed semen samples, and 20 min incubation before motility measurements (in our study) *versus* 4 h [10].

The conclusion that the accumulation is due to thermotaxis is consistent with the relatively small fraction of responsive cell in our assays (Figures 1B and 4). This is because only capacitated spermatozoa are thermotactically responsive [3] and the fraction of capacitated cells is always small in humans [11]–[13] (in this study, 7.6±2.0% on average). As a matter of fact, the extent of sperm accumulation (in this study, up to 5% of the spermatozoa – Figures 1B and 4) is always expected to be somewhat lower than the fraction of capacitated cells. This is because the no-gradient control also contains capacitated cells that arrive coincidentally at the warmer compartment and are subtracted when net thermotaxis is calculated.

### Human Spermatozoa are Thermotactic Over a Wide Temperature Range

One of the surprising findings made in this study is that human spermatozoa are thermotactically responsive over a wide temperature range (29–41°C), much wider than the range considered physiological. The range may be even wider, as evident from the relatively high fraction of responsive cells at the lowest studied temperature (Figure 1B). Another surprising finding is that high temperatures, even temperatures considered detrimental to the sperm function (i.e., temperatures higher than the physiological range), seem to be favored by human spermatozoa. Thus, when human spermatozoa were exposed to a wide temperature range they tended to accumulate at ≥40°C (Figure 2B). Since motility serves as a good indicator of sperm vitality and functionality [14]–[17], an intriguing question is what the physiological significance of sperm accumulation at ≥40°C is. Earlier studies suggested that mild heating may assist fertilization: pre-incubation of human spermatozoa at 40°C was found to improve their fertilizing ability as reflected in the number of pregnancies achieved [18]–[22]. In view of the beneficial temperature effect on the number of pregnancies and the observations made in this study we would not be surprised if, when non-invasive tools to measure the intra-tube temperature become available, it turns out that this temperature in humans is higher than currently believed, perhaps in the vicinity of 40°C.

### Can Spermatozoa Sense a Descending Temperature Gradient?

Generally speaking, two classes of thermotactic behavior have been described: cells that accumulate at some optimal temperature (e.g., *Escherichia coli* [23], *D. discoideum* [24] and *Caenorhabditis elegans* [25] – all known to move both up and down the temperature gradient towards the temperature in which they were grown), and cells that always move up the temperature gradient (e.g., human polymorphonuclear leukocytes, which only respond to ascending thermal gradients [26]). Here we demonstrated that human spermatozoa always move up the temperature gradient in the range tested (Figure 2B) but we do not know whether they can actually sense both ascending and descending temperature gradients or only ascending ones. Indeed, spermatozoa appeared to avoid the cooler compartment (Figure 3), but this could be the outcome of two different mechanisms. One is that capacitated human spermatozoa sense the descending gradient and respond to it by actively turning back to the warmer temperature. The other is that capacitated spermatozoa only sense ascending gradients. According to this possibility they coincidentally reach the cooler compartment and there they sense the ascending temperature gradient and swim back to the warmer compartment.

### The Temperature Sensitivity of Human Spermatozoa in Thermotaxis is Extremely High

The minimal temperature gradient that we could reliably set and measure in this study was 0.014°C/mm. Capacitated spermatozoa thermotactically responded to this gradient perfectly well (Figure 4), suggesting that the threshold is below this value. This threshold is astonishingly shallow. It means that as a spermatozoon swims through its entire body length (46 µm) it can sense and respond to a temperature difference as low as <0.0006°C.

This extremely high temperature sensitivity has physiological significance at three levels. First, such high temperature sensitivity over such a wide temperature range cannot be achieved by a single sensor. Conceivably it could only be achieved by a family of proteins consisting of a large repertoire of thermosensors, each sensitive to a different temperature range, the ranges being superimposed to some extent [5]. Second, since it is implausible that a spermatozoon can sense a temperature difference of <0.0006°C between both its ends, it probably senses the temperature gradient temporally rather than spatially. Temporal sensing may also enable stimulus integration over time and, thereby, stimulus enhancement. This conclusion is consistent with the finding that, during chemotaxis, human spermatozoa apparently sense chemoattractant gradients temporally [27], [28]. And third, if a temperature gradient is established at ovulation in the human Fallopian tube as in the rabbit, and if it is similarly shallow (0.016±0.002°C/mm) [29]–[31], then human spermatozoa can well sense this gradient and respond to it thermotactically.

Human spermatozoa are not alone in their extremely high temperature sensitivity for thermotaxis. Other known examples are root-knot nematodes, which can respond to gradients shallower than 0.0001°C/mm [32], and the pseudoplasmodia of the slime mold *Dictyostelium discoideum*, which can respond to a gradient as small as 0.004°C/mm [33].

## Materials and Methods

### Ethics Statement

The study was approved in a written form by the Bioethics and Embryonic Stem Cell Research Oversight Committee of the Weizmann Institute of Science.

### Media and Chemicals

To handle and capacitate human spermatozoa, we used the commercially available Flushing Medium (MediCult; Jyllinge, Denmark) – a composite of Earl’s balanced salt solution [34], supplemented with sodium pyruvate, synthetic serum replacement, HEPES (23 mM), sodium bicarbonate (12.5 mM), human serum albumin (HSA, 0.1%), penicillin (50000 IU/liter) and streptomycin (50 mg/liter) (pH 7.3–7.4 at 35–37°C). We supplemented the medium with additional 0.2% HSA (Irvine Scientific; Santa Ana, CA, USA), bringing the total HSA concentration to 0.3%. Non-capacitating medium (NCM) was composed of 120 mM NaCl, 4.8 mM KCl, 1.2 mM MgSO_4_, 1.2 mM KH_2_PO_4_, 20 mM sodium lactate, 5 mM glucose, 0.25 mM sodium pyruvate and 40 mM Hepes (pH 7.4). U73122 was obtained from Calbiochem (Gibbstown, NJ, USA), A23187 and polyvinylpyrrolidone (PVP) from Sigma (Munich, Germany), and mouse anti-human CD46:FITC from Serotec (Oxford, UK).

### Spermatozoa

Human semen samples (one sample per experiment) were obtained from nine healthy donors after 3 days of sexual abstinence. Informed consent was obtained in writing from each donor. The semen samples had normal sperm density, motility and morphology (according to World Health Organization guidelines [35]). The semen samples were allowed to liquefy for 30–60 min at room temperature. To obtain sperm samples containing capacitated cells, human spermatozoa were isolated from the seminal plasma by centrifugation (120×g, 15 min, twice) with Flushing Medium supplemented with additional 0.2% HSA. Following this procedure, the sperm samples were adjusted to a concentration of 20–40×10^6^ cells/ml and then incubated for capacitation under an atmosphere of 5% CO_2_ at 37°C for 2 h [11]. The fraction of capacitated spermatozoa was determined from the difference between the levels of acrosome-reacted spermatozoa before and after an acrosome-reaction induction by the Ca^2+^ ionophore A23187, using the acrosomal marker FITC-CD46 as described earlier [5]. To obtain sperm samples mainly containing non-capacitated spermatozoa, fractions of the semen samples (after liquefaction) were diluted 5 times with NCM immediately prior to each experiment. Cell-free seminal fluid was obtained by removing the cells by centrifugation (16,000×g, 5 min, room temperature).

### Thermotaxis Assay

To detect the thermotactic response we used a thermoseparation device (developed and designed by ReproMed Ltd, Israel) consisting of two basic units: an electrical unit, which creates and maintains a temperature gradient by heating one end and cooling the other end, and a Lucite tube composed of two compartments for the separation process (Figure 1A; see also [5]). One compartment was filled with human spermatozoa (30–50×10^6^ cells/ml; 37°C) that had been allowed to capacitate for 2 h, or with semen that was diluted 5 times with NCM (37°C). The other compartment (termed the target compartment) was filled with the same medium (Flushing medium, 37°C) but with no spermatozoa, or with cell-free seminal fluid diluted 5 times with NCM (37°C), respectively. The tube was closed with two Lucite stoppers, inserted into the thermoseparation device, and then it was incubated within the device for 15 min. This allowed the formation of a linear temperature gradient within the tube (see below) and the migration of spermatozoa from one compartment to the other. Finally, the spermatozoa were collected from the target compartment and counted by a Z2 Coulter counter analyzer (Beckman Coulter Inc.; Miami, FL, USA; counting accuracy: ±1%). As a negative control for coincidental migration to the target compartment, the same procedure was repeated, but this time with a constant temperature (no gradient) along the tube, with both compartments at the same temperature.

### Temperature-dependent Sperm Accumulation

To measure human sperm distribution within a temperature gradient we used a linear drinking straw (made of plastic; 5 mm inner diameter) that was fitted to our thermoseparation device both in width (adjusting with masking tape to a 7 mm outer diameter) and length (38 mm; Figure 2B). The straw was filled with human spermatozoa (20×10^6^ cells/ml) that had been allowed to capacitate for 2 h, and closed by two silicone stoppers. To reduce fluid movements within the straw, the Flushing Medium also contained 3.5% PVP, found not to harm cell motility and the capacitation level [28]. (Increased viscosity was essential for this experiment because, in the absence of PVP, the distribution of cells along the straw was roughly homogenous – data not shown.) The straw was incubated in the device for 20 min. As soon as the straw was removed from the device, it was placed in liquid nitrogen in order to preserve the cell distribution in the straw. The frozen straw was cut into 8 equal parts and the number of spermatozoa in each part was counted by a Coulter counter.

### Linearity of the Internal Temperature Gradient

The temperature gradient provided by the thermoseparation device was linear according to the manufacturer’s specifications. To measure the actual temperature within the tube and the straw and to determine the linearity of the internal gradient, a thin thermocouple (additional to the thermocouples at both ends of the thermoseparation device) was inserted into them through a tiny hole in the tube’s or straw’s stopper. The temperature at sequential locations within the tube and straw was measured employing an independent thermometer. Measurements carried out after the 15 min incubation period indicated that the inner temperature was equal to the outer temperature at the warmer end of the tube/straw. However, depending on the temperature range, it was higher at the cooler end: up to 1°C or 3°C difference in the tube or straw, respectively. The inner temperature measurements along the tube and straw confirmed the existence of an internal linear temperature gradient (Figure S1). Furthermore, the linearity of the internal gradient was verified experimentally for each of the temperature gradients tested in this study.

### Motility Assays

To determine the sperm motility, different sperm samples, pre-incubated for capacitation, were incubated (in the presence and absence of 3.5% PVP) for 15–20 min at the indicated temperatures. Each sperm sample (10 µl containing 5×10^6^ cells/ml) was subsequently transferred to a bright-line haemocytometer on top of a thermostated phase-contrast microscope (both kept at the same measuring temperature) and the sperm motility was immediately followed with an objective ×10 and a high resolution CCD camera (Migvan), connected to a computer equipped with a video capture external card (DVD EZMaker, AverMedia).

### Analysis of Sperm Kinetic Parameters

The analysis of sperm kinetic parameters was carried out by homemade software that collected in real time digitized data from four 20 s time segments (80 s in total) at 25 frames/s. It provided the coordinates of the center of the head of each spermatozoon in each frame and analyzed the commonly used kinetic parameters [36], [37] of each spermatozoon for up to 20 s track length: VCL, curvilinear velocity (the time-average velocity of the sperm head along its actual sampled path, calculated by summing the incremental frame-to-frame distances made by the sperm head along the path and dividing by the total time of the track); VSL, straight-line velocity (the time-average velocity of the sperm head along a straight line from its first position to its last position, termed also progressive velocity); LIN, percent linearity (the ratio VSL/VCL multiplied by 100); STR, percent straightness (the ratio between the straight line from the first point on the smoothed path to the last point on this path and the total distance along the smoothed path, multiplied by 100); MOT, percent motile cells (only spermatozoa with path velocity >5 µm/s were considered motile); HYP, percent hyperactivated cells (defined herein as cells having VCL ≥70 µm/s, LIN ≤30% and amplitude of lateral head displacement ≥7 µm [38]). For each determination 300–1800 cells were analyzed, and each such analysis was repeated in duplicate with 3 sperm samples. The above-mentioned conditions for motion analysis followed the guidelines for CASA instruments [39], excluding the recommended video framing rate for fast-swimming cells (60 frames/s).

### Statistical Analysis

InStat 3 software package (Graph Pad Software; San Diego, CA, USA) was used for statistical calculations. The significance of the difference between the treatments was calculated by a contrast *t*-test or by a repeated measures or one-way ANOVA test with proper post-tests, as indicated.

## Supporting Information

Figure S1 **Temperature gradient within a Lucite tube and a straw.** The temperatures were measured 2–3 times using thin thermocouples connected to an independent thermometer. Zero represents the colder end of the tube or straw. The results are presented as mean ± SEM. The straight lines are linear fits (R^2^ = 0.99).
(TIF)

Figure S2 **Dependence of sperm accumulation on the magnitude of the temperature difference (constant temperature in target compartment).** The temperature of the target compartment was 41°C in all runs. The temperature differences shown in the abscissa were measured by the thermocouples at both ends of the tube (externally to the tube). Net accumulation was calculated by subtracting the no-gradient control accumulation from the sperm accumulation in a temperature gradient. The results are the mean ± SEM of 10 experiments.
(TIF)
