# Supplementary figures and images for: Thermotaxis of Human Sperm Cells in Extraordinarily Shallow Temperature Gradients Over a Wide Range
Source: PLoS One. 2012 Jul 25;7(7):e41915. doi: 10.1371/journal.pone.0041915 (PMC3405043; doi:10.1371/journal.pone.0041915)

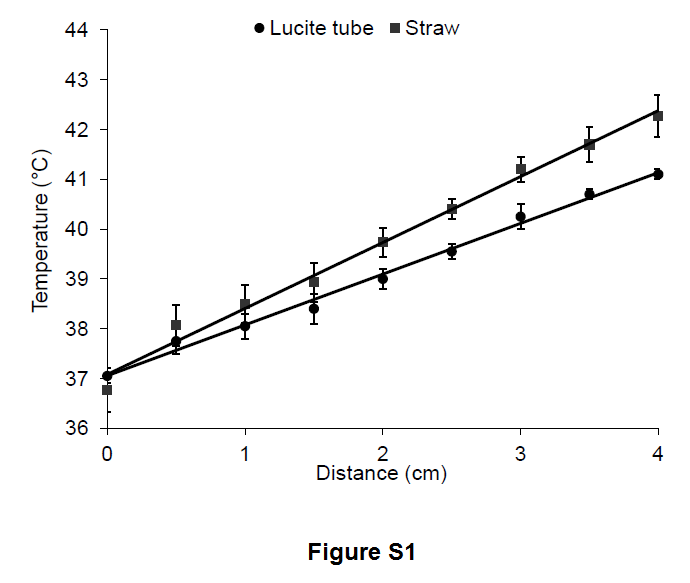

Supplement: Figure S1 — Temperature gradient within a Lucite tube and a straw. The temperatures were measured 2–3 times using thin thermocouples connected to an independent thermometer. Zero represents the colder end of the tube or straw. The results are presented as mean ± SEM. The straight lines are linear fits (R2 = 0.99). (TIF) [file pone.0041915.s001.tif]

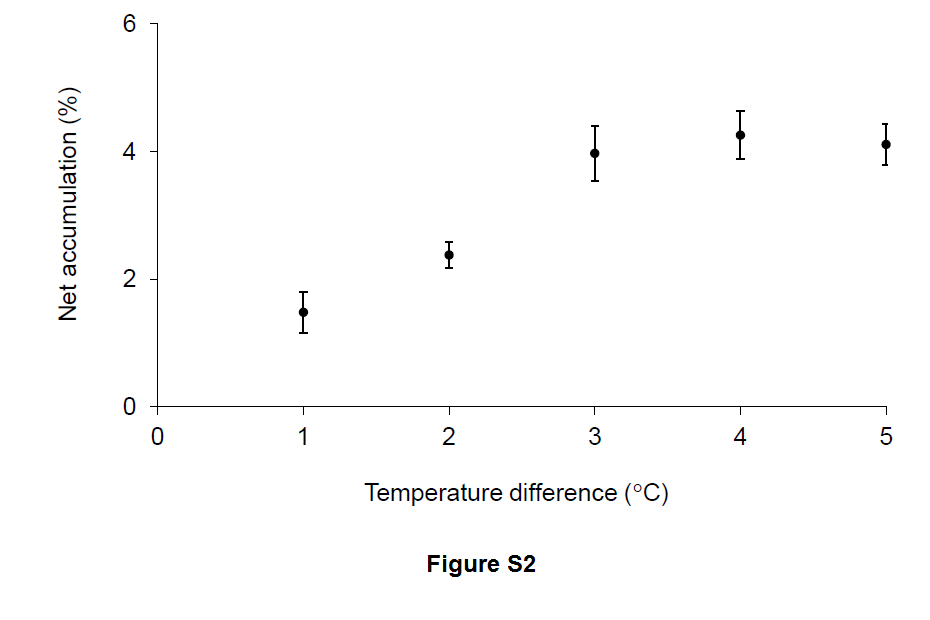

Supplement: Figure S2 — Dependence of sperm accumulation on the magnitude of the temperature difference (constant temperature in target compartment). The temperature of the target compartment was 41°C in all runs. The temperature differences shown in the abscissa were measured by the thermocouples at both ends of the tube (externally to the tube). Net accumulation was calculated by subtracting the no-gradient control accumulation from the sperm accumulation in a temperature gradient. The results are the mean ± SEM of 10 experiments. (TIF) [file pone.0041915.s002.tif]
